# Supplementary material for: Human RBM3 protein is prone to form neuronal aggregates opposed by the proteasome
Source: Biol Open. 2026 Jan 2;15(1):bio062179. doi: 10.1242/bio.062179 (PMC12805641; doi:10.1242/bio.062179)
Supplement: Supplementary information [file biolopen-15-062179-s1.pdf]

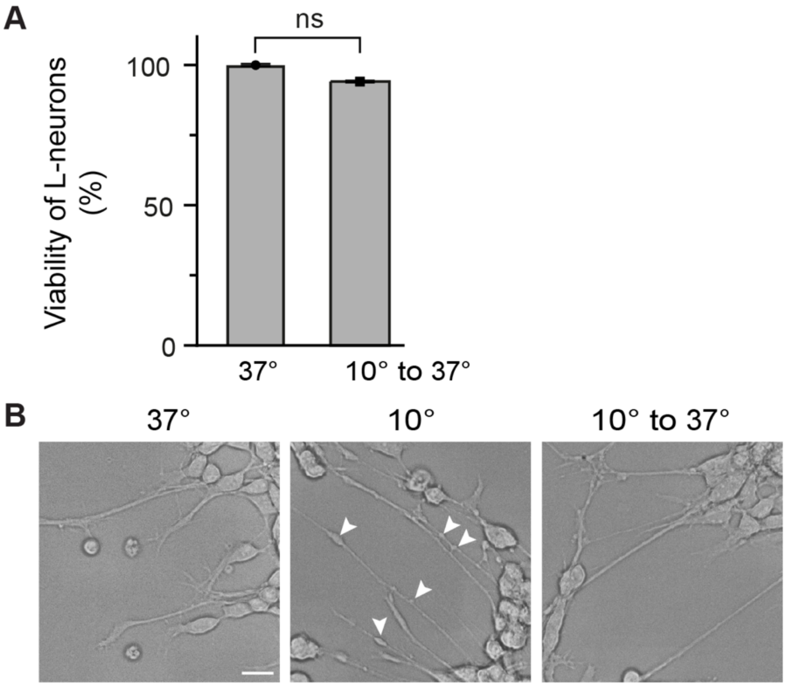

Fig. S1. The neurons survive deep hypothermia. A. Viability of L-neurons tested by the CCK-8 assay in cells incubated at 10°C for 16 h and rewarmed at 37°C for 1 h. Unpaired two-tailed t test was used to calculate statistical significance. $p = 0.94$ (ns). **B.** Brightfield image of L-neurons showing the neurite morphology at 37°C, 10°C (incubation for 16 h), and rewarmed at 37°C for 1 h. Arrowheads point to the bead-like swellings along neurites that disappear following rewarming. Scale bar: 20 µm.

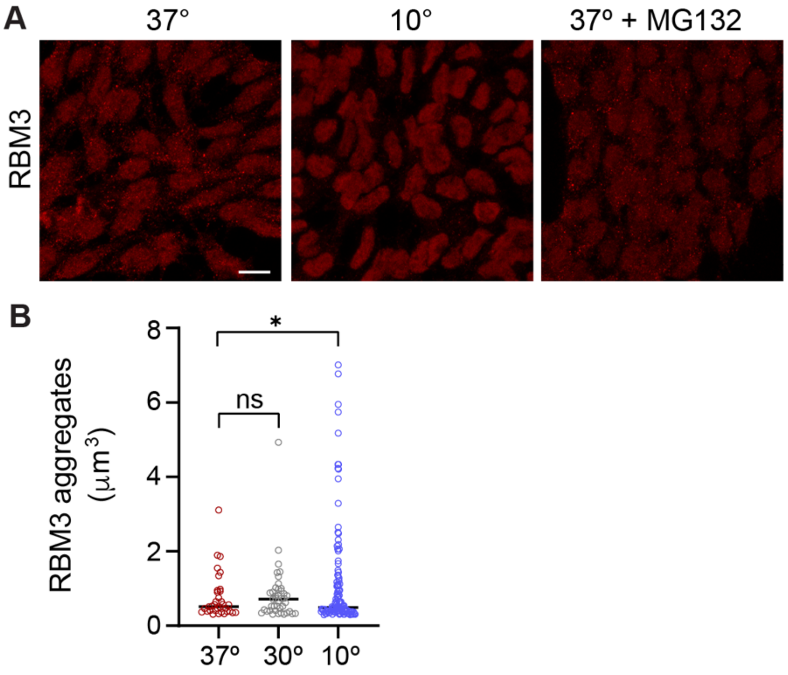

Fig. S2. Differentiation-related aggregation of RBM3. A. Immunodetection of RBM3 in undifferentiated LUHMES cells treated as indicated. MG132 was added at 20 μM for 6 h. Scale bar: 10 μm**. B.** Quantification of RBM3 aggregates in L-neurons. The neurons were incubated at 37°C, 30°C, or 10°C, and the aggregates were scored per 300 cells/condition from 3 biological replicates, with the lower size cutoff of 0.3 μm^3. The number of RBM3 aggregates per cell: 0.11 at 37°C; 0.13 at 30°C, and 0.39 at 10°C. Welch's t test was used for two-condition comparisons. 10ºC vs. 37ºC, $p = 0.027$ (*); 30ºC vs. 37ºC, $p = 0.646$ (ns).

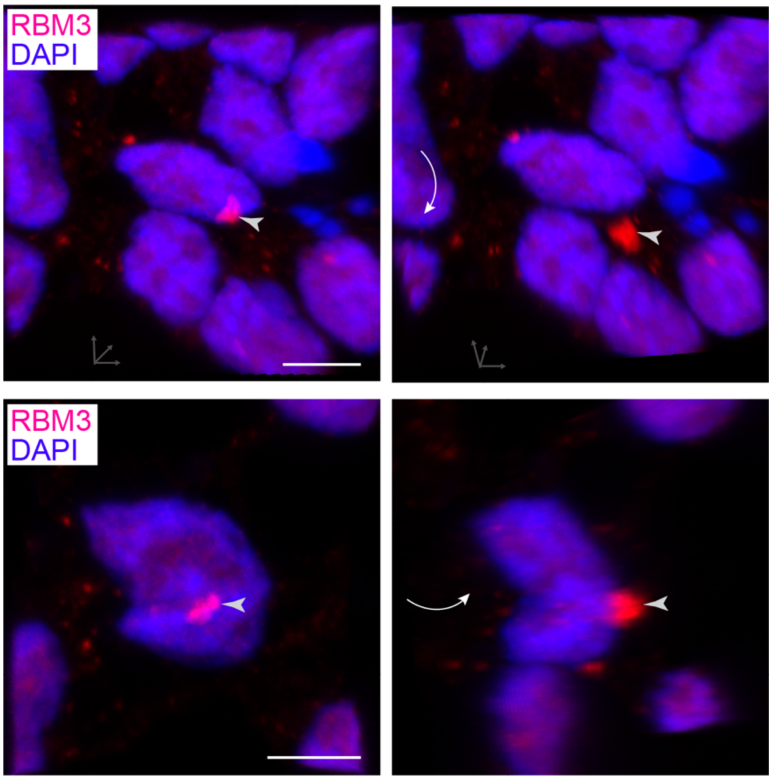

Fig. S3. RBM3 aggregates in the neuronal body are cytoplasmic. Shown are 3D images of L-neurons stained with RBM3 antibodies and DAPI. Left: The images show some RBM3 aggregates seemingly adjacent to the nuclei (arrowheads). Right: Rotating the images reveals, however, that these aggregates are outside the nuclei, i.e. in the cytoplasm of neuronal soma. Scale bars: 5 µm.

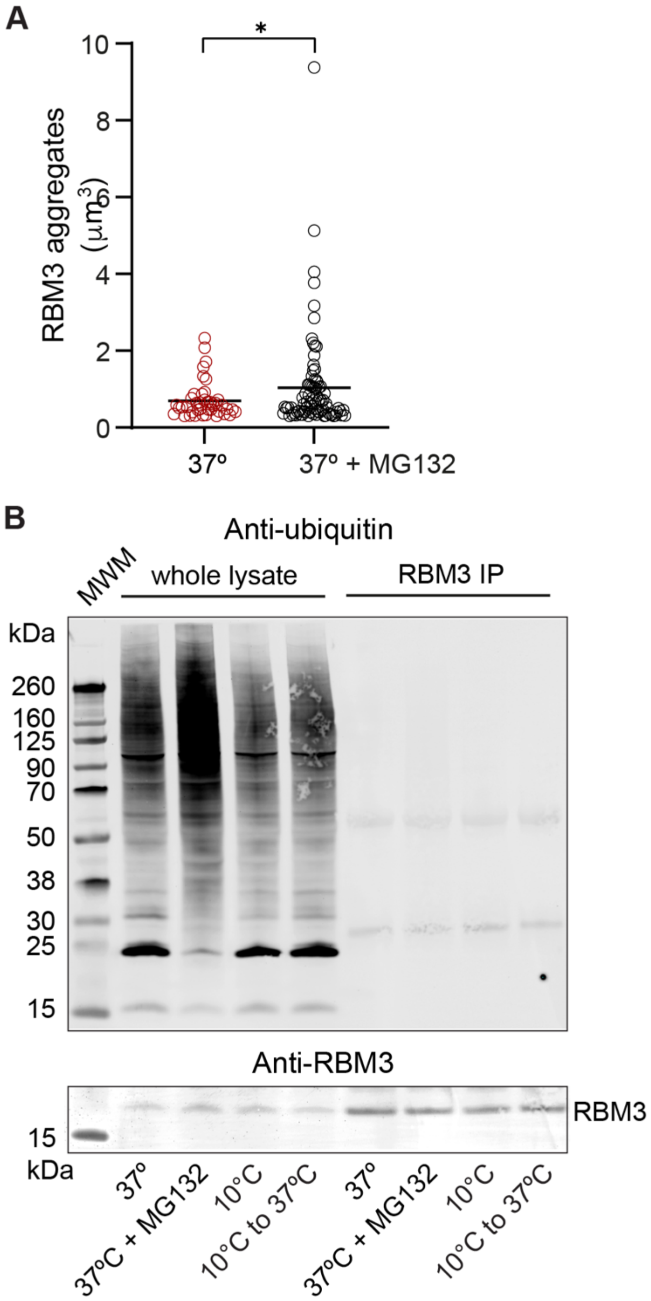

Fig. S4. RBM3 aggregates form at 37ºC upon proteasomal inhibition in the absence of RBM3 ubiquitination. A. Quantification of RBM3 aggregates in L-neurons. The neurons were incubated at 37°C, 30°C, or 10°C, and the aggregates were scored per 260 cells/ condition from 3 biological replicates, with the lower size cutoff of 0.3 μm^3. The number of RBM3 aggregates per cell: 0.16 at 37°C; 0.31 at 37ºC upon MG132 treatment (20 µM, 6 h). Welch's t test was used for comparison, $p = 0.038$ (*). **B.** Western blot analysis of whole lysates and RBM3 immunoprecipitates (IP) from L-neurons, using anti-ubiquitin (upper panel) or anti-RBM3 (lower panel) antibodies.

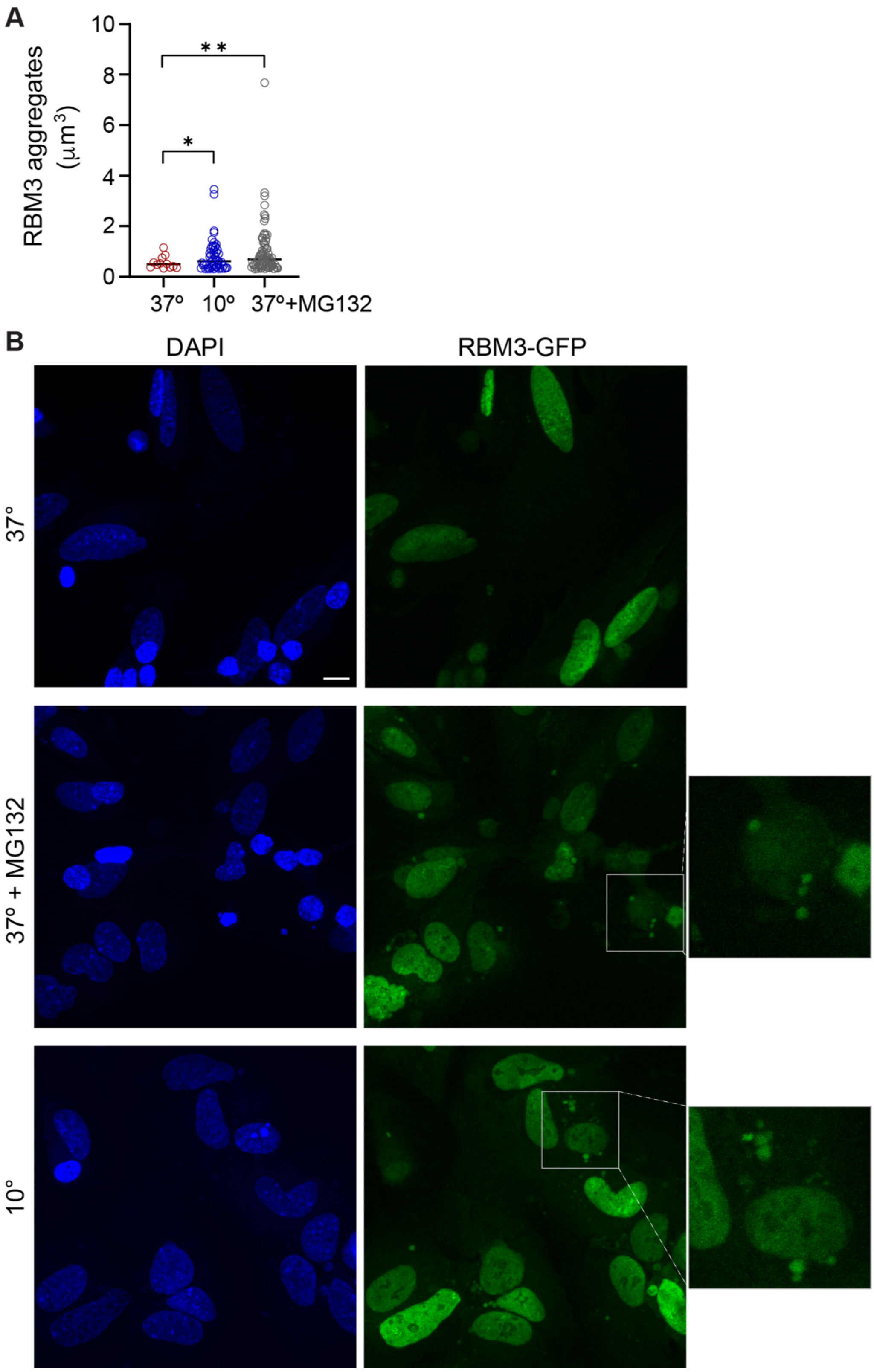

Fig. S5. RBM3 aggregates form in SH-SY5Y cells and can be visualized upon GFP-tagging of RBM3. A. Quantification of RBM3 aggregates in wild-type SH-SY5Y cells, with the size cutoff of 0.3 µm^3. Around 75 cells were scored per condition from 3 biological replicates. The number of RBM3 aggregates per cell: 0.16 at 37°C; 0.68 at 10°C and 1.02 upon MG132 treatment (15 µM, 5 h). Welch's t test was used for two-condition comparisons. 37°C vs. 10°C p = 0.024 (*); and 37°C vs. 37ºC+MG132, p = 0.001 (**). **B.** SH-SY5Y neurons stably expressing RBM3-GFP form aggregates at 10°C or at 37ºC upon MG132 treatment (15 µM, 5 h). The cells were fixed with 4% PFA prior to imaging. Boxed areas are magnified on the right. Scale bar: 10 µm.

Biology Open • Supplementary information

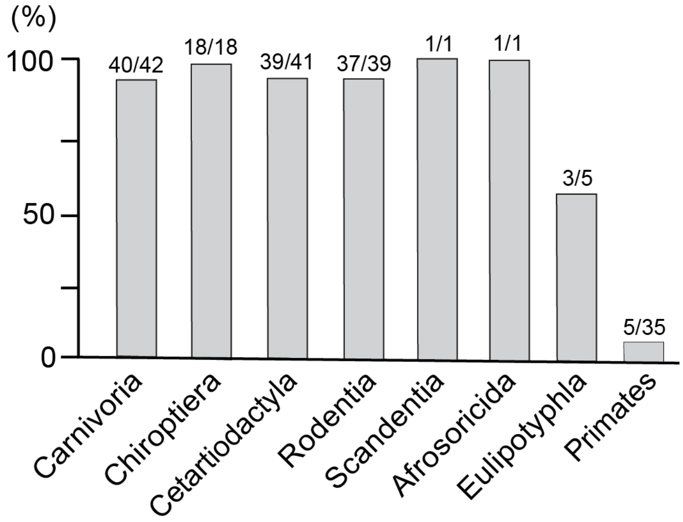

Fig. S6. The proportion of species within different mammalian orders containing both Arg^{135+} and Arg^{135-} isoforms. Based on available human RBM3 orthologs in NCBI Eukaryotic Genome annotation pipeline. Note that in primates, where the hibernation/torpor is uncommon, the Arg^{135-} isoform is rare compared with other orders.

Biology Open • Supplementary information

Order	Species	Isoform	Aligned ROI
Carnevoria	*Meles meles* (European badger)	Isf.1	R D Y G G - S Q G G
		Isf.2	R D Y G G R S Q G G
	Ursus arctos (Brown bear)	Isf.1	R D Y G G R W V A K
		Isf.2	R D Y G G - S Q G G
		Isf.3	R D Y G G R S Q G G
Chiroptiera	*Artibeus jamaicensis* (Jamaican fruit bat)	Isf.1 and 3	R D Y G G R S Q G G
		Isf.2 and 4	R D Y G G - S Q G G
	Eptesicus fuscus (Big brown bat)	Isf.1	R D Y G G R S Q G G
		Isf.2	R D Y G G - S Q G G
	Myotis lucifugus (Little brown bat)	Isf.1	R D Y G G R S Q G G
		Isf.2	R D Y G G - S Q G G
Rodentia	*Ictidomys tridecemlineatus* (Thirteen-lined ground squirrel)*	Isf.1	R D Y S G R S Q G G
		Isf.2	R D Y S G - S Q G G
	Cricetulus griseus and *Mesocricetus aurus* (Chinese hamster and golden hamster)	Isf.1	R D Y S G R S Q G G
		Isf.2	R D Y S G - S Q G G
	Phodopus roborovskii (Rodorovski dwarf hamster)	Isf.1	R D Y S G R S Q G G
		Isf.2	R D Y S G - S Q G G
	Mus musculus (House mouse)	Isf.1	R D Y S G R S Q G G
		Isf.2	R D Y S G - S Q G G
	Peromyscus maniculatus bairdii (Prairie deer mouse)	Isf.1	R D Y S G R S Q G G
		Isf.2	R D Y S G - S Q G G
Scandentia	*Tupaia chinensis* (Chinese tree shrew)	Isf.1	R D Y S G R S Q G G
		Isf.2	R D Y S G - S Q G G
Eulipotyphla	*Sorex araneus* (Common shrew)	Isf.1	R D Y S G R S Q G G
		Isf.2	R D Y S G - S Q G G
		Isf.3	R D Y G G R S Q G G
	Suncus etruscus (Etruscan shrew)		R D Y G G R S Q G G
	Erinaceus europaeus (European hedgehog)		R D Y G G R N Q G G
Afrosoricida	*Echinops telfairi* (Lesser hedgehog tenrec)	Isf.1	R D Y G G R S Q G G
		Isf.2	R D Y G G - S Q G G
Primates	*Microcebus murinus* (Gray mouse lemur)	Isf.1	R D Y G G R S Q G G
		Isf.2	R D Y G G - S Q G G

Arg^{135}

Fig. S7. Alignment of RBM3 proteins from known hibernating mammals. The species are colour-coded according to mammalian orders. *4 other rodent species have identical proteins. Note that all but two species express the Arg^{135-} RBM3 isoform (apart from the Arg^{135+} variant).

Biology Open • Supplementary information

Table S1. Assembly of mammalian hibernators. Columns 1 and 2 are based on Geiser et al, 2013 (Table 1). Column 3 shows search words used in the RBM3 ortholog dataset and column 4 search results; if there were many hibernators within a mammalian order, the indicated species were chosen for Figs 7 and S7.

**Family/Order**	**Description**	**Modified search in dataset**	**Species found**
Egg-laying mammals (Monotremata)	Echidnas hibernate in many areas of Australia.	Only placental present in the datset	None
Opossums (Didelphimorpha)	Several small south American opossums enter daily torpor.	Didelphimorpha, opossum,	None
Shrew-opossum (Microbiotheria)	The Monito del monte hibernates in southern Chile.	Microbiotheria, australidelphia	None
Carnivorous marsupials (Dasyuromorphia)	Many dasyurids <1 kg enter daily torpor.	Dasyuromorphia, australidelphia	None
Marsupial moles (Notoryctemorphia): Marsupial moles are heterothermic. Possums (Diprotodontia)	Pygmy-possums and feathertail gliders hibernate; other gliding possums use daily torpor.	Notoryctemorphia, possums, marsipual, marsipual mole	None
Tenrecs, golden moles (Afrosoricida)	Tenrecs hibernate or express torpor; golden moles are heterothermic.	Tenrec, mole, golden mole, afrosoricidam	Lesser hedgehog, tenrec
Elephant shrews (Macroscelidea)	Short-term hibernation and daily torpor.	Elephant shrew, macroscelidea	None
Armadillos (Edentata, Cingulara)	Hibernation in at least one species of armadillo.	Edentata, cingulara, armadillo	While other family members hibernate, this has not been shown specifically for the Nine-Banded armadillo, which is why we did not include it.
Rodents (Rodentia)	Hibernation and daily torpor are widespread among rodents.	Rodentia, rodents, Ground squirrel, hamster, mouse, deer mouse	Ground squirrel and hamsters are known hibernators, deer mouse also hibernate, and mouse enter torpor when starved and cooled.
Primates (Primates)	Hibernation in fat-tailed lemurs and other small lemurs, daily torpor in bushbabies.	Primate, lemur, prosiminan	The gray mouse lemur was chosen due to abundant literature.
Insectivores (Insectivora, Lipotyphla	Hedgehogs hibernate; shrews use daily torpor.	Insectivore, Hedgehog, Shrew, Eulipotyphla	- Erinacues europaues,<br>- Suncus etruscus<br>- Sorex araneu<br>- Sorex fumes,

Biology Open • Supplementary information

			- Talpa occidentalis 5 species found, all known to hibernate. Sorex araneu, Erinacues and suncus etruscus was chosen as representative for Figure S7.
Bats (Chiroptera)	Hibernation in many insectivorous bats; small fruit bats use daily torpor.	Chiroptera, bats, Fruit bats	42 species, chose a few examples that are known to hibernate.
Carnivores (Carnivora)	Shallow hibernation in bears and badgers; shallow torpor in skunk.	Carnivoria, bear, skunk, badger	Brown bear, european badger

Biology Open • Supplementary information
